# Mesoscale eddy-strengthened deep-sea topographic Rossby waves in the southwestern South China Sea

**DOI:** 10.1038/s41598-024-62040-z

**Published:** 2024-05-18

**Authors:** Wenzhuo Wang, Zhifei Liu, Yulong Zhao, Baozhi Lin, Xiaodong Zhang, Jingwen Zhang, Jiaying Li, Junyuan Cao, Hongzhe Song

**Affiliations:** State Key Laboratory of Marine Geology, Tongji University, Shanghai, China

**Keywords:** Hydrology, Ocean sciences

## Abstract

Topographic Rossby waves (TRWs) dominate the low-frequency variability of deep ocean currents and play a crucial role in energy exchange and material mixing. On the continental slope of the southwestern South China Sea, a deep-water mooring was deployed to observe TRWs for a period of ~ 40 days. The TRWs, with a wavelength of 109 km, account for 41.3% of the subinertial variations. A ray-tracing model was applied to investigate the propagation and energy source. The results showed that the TRWs propagated from the northeast of the mooring location and were most likely caused by the mesoscale eddy disturbances off the Vietnam coast. This study provides a new perspective on examining the impact of mesoscale eddies off Vietnam on abyssal currents.

## Introduction

Topographic Rossby waves (TRWs) are generated by cross-isobathic motion due to the transformation of water columns over the sloping submarine topography, under the conservation of potential vorticity^1^. TRWs have periods ranging from tens to hundreds of days, and they propagate with shallower water to their right^2^. It is understood that TRWs dominate the low-frequency variability of deep currents^3^ and regulate deep circulation^4^. In this way, TRWs play a crucial role in deep-sea energy exchange, as well as substance and heat transport^5^, eventually exerting an impact on climate change^6^.

According to previous studies, energetic TRWs activities have been found around the Gulf Stream region^5,7–10^ since their first observation^11^. It shows that TRWs are forced by the deep meandering Gulf Stream and warm core rings, with Loop Current eddies transferring energy^5,10,12^. Apart from the Gulf of Mexico, TRWs with a period of 3–15 days were observed in the water volume transport from the Japan Basin to the Yamato Basin^13^. In the Pacific Yap-Mariana Junction, TRWs were found to reach a maximum magnitude of 45 cm/s and they were generated by surface eddies and instability of the background flow^4^. Based on multiyear mooring observation data, Shin et al.^14^ explained that the longer intraseasonal fluctuation (30–50 days) of the Dokdo Abyssal Current was caused by the TRWs. Observations in the Arctic Ocean suggested that TRWs are sources of subinertial currents that propagate with shallow water on the right^15^.

The South China Sea (SCS) is the largest marginal sea in the northwestern Pacific Ocean, and it provides suitable conditions for TRWs generation due to its complex submarine topography and seamount groups. The deep circulation of the SCS is promoted cyclonically by the north Pacific deep water through the Luzon Strait^16^, which is the only deep channel connecting the SCS to the Pacific Ocean^16–19^. Shu et al.^20^ were the first to report persistent TRWs in the SCS, with a period of 9–14 days and a wave length of approximately 82 km. and they found that the energy of TRWs over the steep topography was comparable to that of internal tides. Westwards-propagating TRWs with an oscillation period of approximately 14.5 days and a group velocity of ~ 10 cm/s were also observed around the Dongsha Islands in the northern SCS^21^. These TRWs accounted for more than 40% of the deep current variations^21^. TRWs generated by surface mesoscale eddies with a period of ~ 65 days were also observed in the deep SCS basin, which also propagated westward^22^.

**Figure 1:**
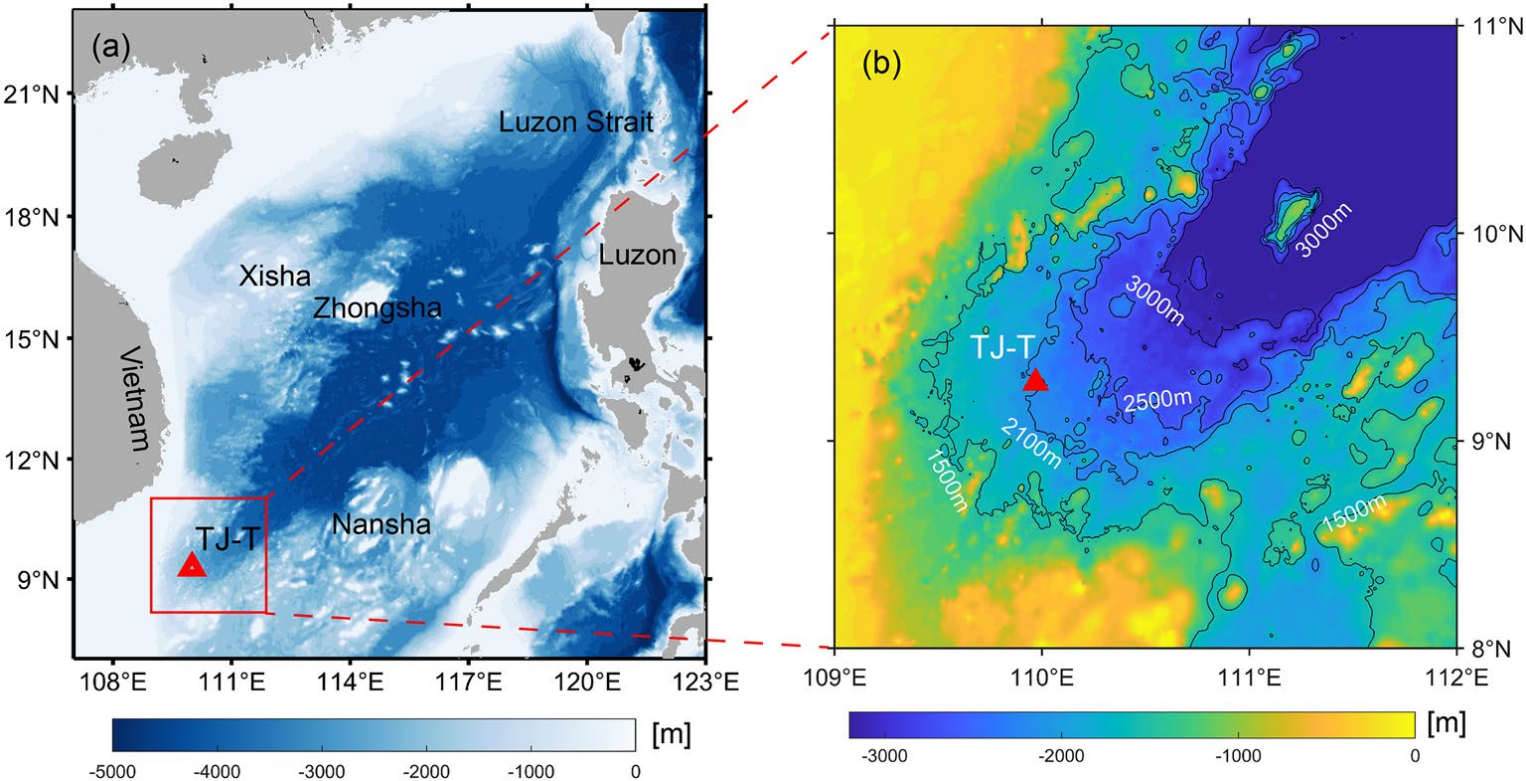
Topography of the South China Sea (SCS) and location of the mooring TJ-T. (**a**) Topography of the SCS; (**b**) zoom-in bathymetry of the continental slope of the southwestern SCS. The red triangle represents the location of mooring TJ-T. The maps were generated by MATLAB R2019b (https://ww2.mathworks.cn/) with M_Map (a mapping package, https://www.eoas.ubc.ca/~rich/map.html).

Previous investigations on TRWs in the SCS have focused mainly on the northern and central basins, where the Kuroshio intrusion and seamount topography create a typical active dynamic environment. In contrast, due to a lack of observations, little is known about the characteristics and energy sources of TRWs in a weaker dynamic background, e.g., the southwestern basin of the SCS, and to what extent they contribute to local intraseasonal fluctuations. In this study, a subaqueous mooring (TJ-T) was deployed on the continental slope of the southwestern SCS from June 2020 to October 2021 (Fig. 1), revealing the spatial and temporal variations of TRWs and attempting to understand their energy sources.

## Results

### Characteristics of deep-current variability

**Figure 2:**
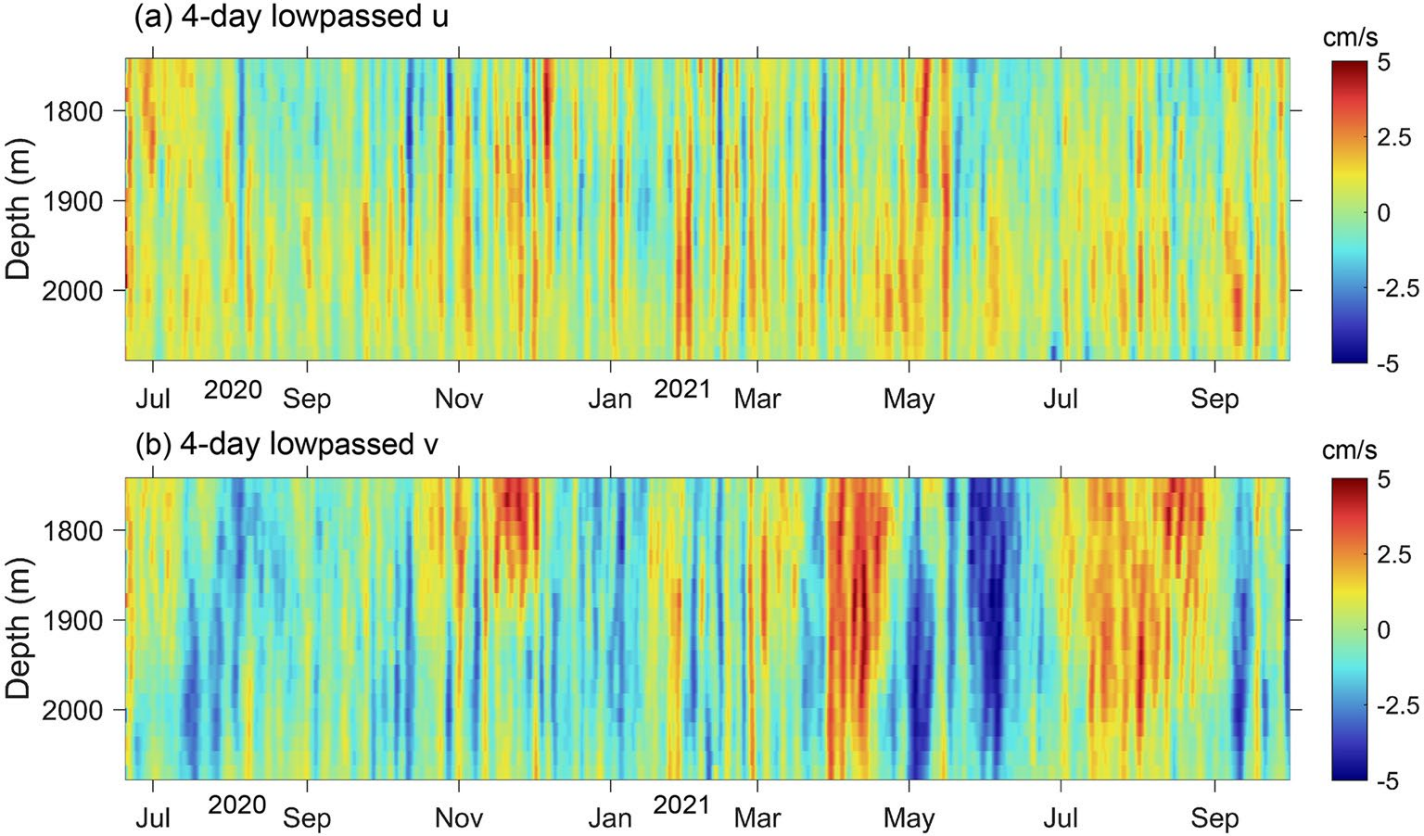
Observed horizontal velocity at the mooring TJ-T in the southern SCS. Time series of 4 days lowpassed zonal velocity u (**a**) and meridional velocity v (**b**) profiles from Acoustic Doppler Current Profiler (ADCP) observations.

The mooring TJ-T is located at the nook of the southwest SCS sub-basin, where the direction of the thalweg is nearly north–south oriented (Fig. 1). Therefore, our analysis was all based on the meridional velocity component (v) and zonal velocity component (u). A Butterworth low-pass filter with a cut-off frequency of 0.25 cycles per day (cpd) was applied to the raw velocity to remove the signal of tides and near-inertial oscillations (1/3.02 cpd). The profiles of lowpassed velocity are shown in Fig. 2. The zonal velocity u generally flowed eastward with a time-average value of about 0.3 cm/s, which agrees with the deep cyclonic circulation in the SCS. The large standard deviations relative to the mean value of velocity indicate the strong variabilities (Table 1). It is clear that the meridional component dominated the current fluctuation, with significant intraseasonal variabilities (days to dozens of days). The strongest fluctuation occurred between April 2021 and June 2021, with long periods throughout the observed layers.

**Table 1:** Mean values and standard deviations of the velocity components at depths of 1742 m, 1854 m, 1966 m, and 2078 m at the mooring TJ-T.

Water depth [m]	$\overline{{\text{v}} }$ [cm/s]	σ(v) [cm/s]	$\overline{{\text{u}} }$ [cm/s]	σ(u) [cm/s]
1742	− 0.02	1.85	− 0.04	1.38
1854	− 0.01	1.88	0.09	1.38
1966	− 0.41	1.61	0.72	1.20
2078	0.10	0.99	− 0.06	1.09

**Figure 3:**
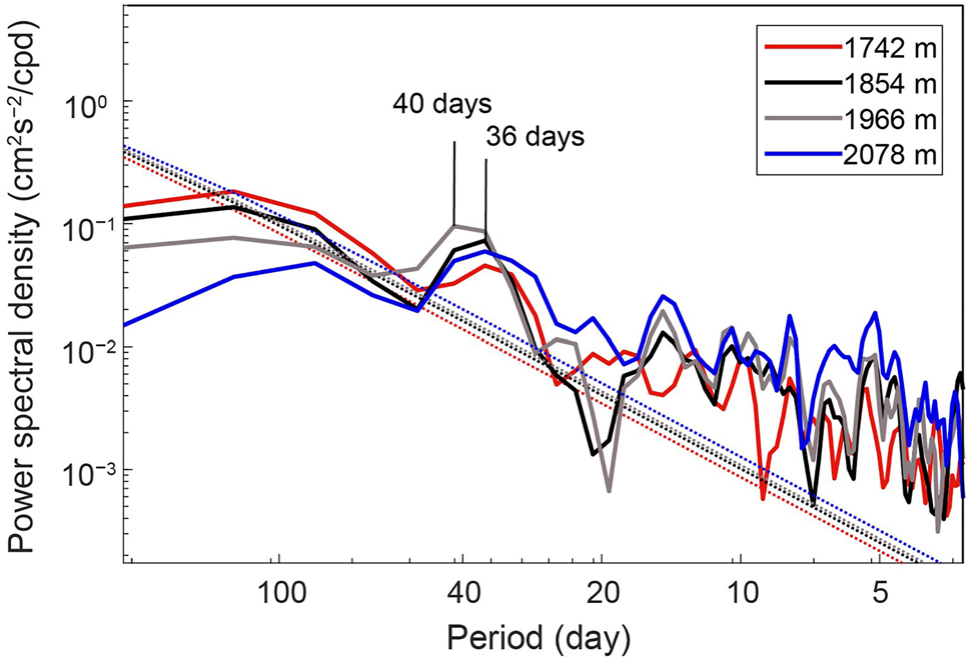
Power spectra of meridional velocity at 1742 m, 1854 m, 1966 m, and 2078 m water depth from the mooring TJ-T observations between June 20, 2020 and October 1, 2021. Dashed lines represent significant levels of 95%.

**Figure 4:**
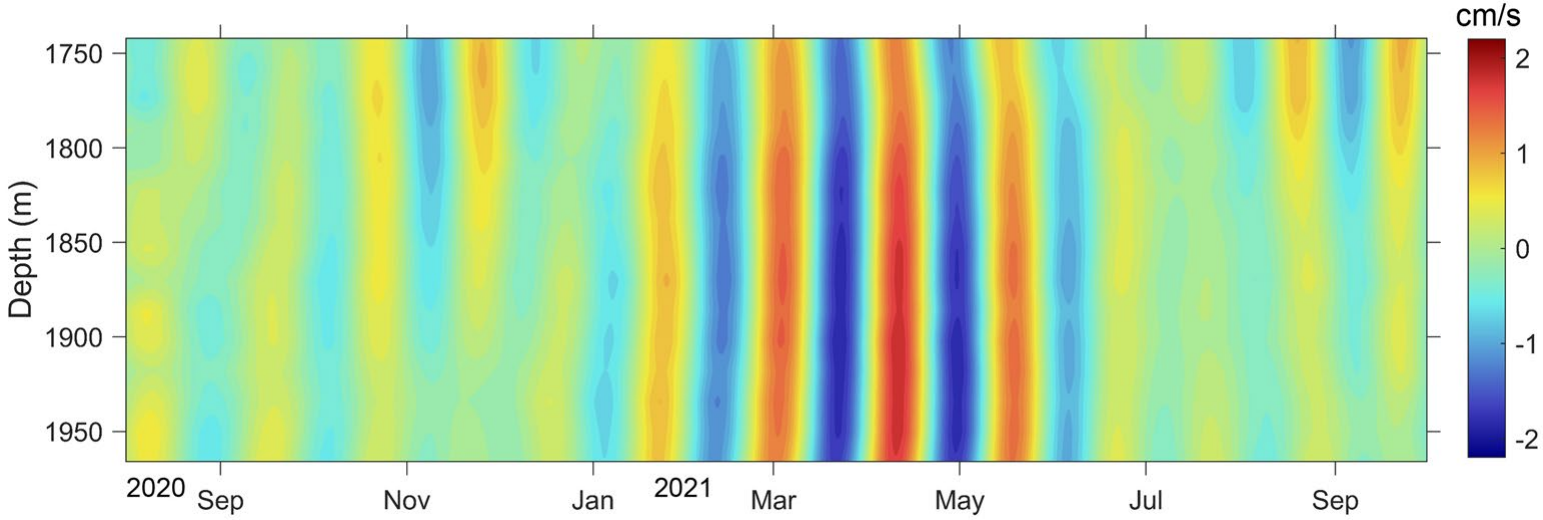
Time series of meridional velocity (v) profile after 30–50 day band-pass filtered from the mooring TJ-T in the southern SCS.

To determine the dominant periods, the power spectra of the subinertial v were calculated (Fig. 3). Significant peaks of around 40 days were observed in all the 4 layers (1742 m, 1854 m, 1966 m, 2078 m). This fluctuation contributed approximately 41.3% of the subinertial current variabilities throughout the observation period. It intensified since late March and dominated the enhancement of subinertial current, as shown in Fig. 4. The fluctuation strengthened with increased water depth from 1742 to 1966 m from March to May 2021, when the ~ 40 days cycles were the strongest. In addition, no apparent vertical phase propagation was observed, suggesting its coherent nature.

### Features of TRWs

The power spectrum reveals a significant intraseasonal fluctuation with a period of approximately 40 days in the meridional direction. The band-passed current showed that this period was significant from March to May 2021 (Fig. 4). Considering that no apparent vertical phase propagation was observed, this ~ 40 days oscillation is very likely caused by the TRWs^20,23,24^.

Based on the linear quasi-geostrophic potential vorticity equation, the TRW dispersion relation can be introduced as follows^1,25^:

1 $$\lambda_{d}^{2} = \left( {k^{2} + l^{2} + \frac{\beta k}{\omega }} \right)\left( {\frac{N}{{f_{0} }}} \right)^{2} ,$$

2 $$\lambda_{d} \,tanh\left( {\lambda_{d} {\text{H}}} \right) = \frac{{N^{2} }}{{\omega f_{0} }}\left( {k\frac{{\partial {\text{H}}}}{\partial y} - l\frac{{\partial {\text{H}}}}{\partial x}} \right),$$

where $\omega$ is the TRW frequency, (*k, l*) are the zonal and meridional wavenumbers, $f$ is the Coriolis parameter and is calculated as $f={f}_{0}+\beta y$ (where $\beta =\partial f/\partial {\text{y}}$), H is the water depth, $1/{\lambda }_{d}$ is the vertical trapping scale of the wave, and $N$ is the Brunt-Väisälä frequency. At the mooring location, ${f}_{0}$ is 2.3518 × 10^−5^ s^−1^, and the topographic gradient $\left|\nabla {\text{H}}\right|$ is ~ 0.01 after applying a 111 km smooth, which is larger than the local internal Rossby deformation radius *L*_D_ (*L*_D_ = $N$ H/$f$)^23^. The topographic beta $\beta_{top} = \frac{{f_{0} \left| {\nabla {\text{H}}} \right|}}{{\text{H}}} = 1.05\, \times \,10^{ - 10}$ m^−1^ s^−1^, which is much greater than the planetary beta *β* = 2.26 × 10^−11^ m^−1^ s^−1^. Therefore, the term *β* is omitted and the TRW dispersion relation is written as:

3 $$\omega = \frac{{ - k\left| {\nabla {\text{H}}} \right|N}}{{{\mathbf{K}}\,coth\left( {\frac{{N{\varvec{K}}{\text{H}}}}{f}} \right)}},$$

where ${K}^{2}={k}^{2}+{l}^{2}$. For short waves, NKH/$f$ > 1 and coth(NKH/$f$) ≈ 1. Then Eq. 3 can be simplified to:

4 $$\omega = - \frac{{k\left| {\nabla {\text{H}}} \right|N}}{{\mathbf{K}}} = \left| {\nabla {\text{H}}} \right|N\,sin\,\theta ,$$

where *θ* is defined as the clockwise angle that the wavenumber vector (K) makes with the direction of topographic gradient $\left| {\nabla {\text{H}}} \right|$, or alternatively, as the angle between the velocity vector and isobaths^11,20^. This angle ranges between 0 and $\pi$. The group velocity, denoted as ${c}_{g}=(\partial \omega /\partial {\text{k}},\partial \omega /\partial l)$, is perpendicular to the wavenumber vector. Consequently, shallow water is located on the right side of the group speed^10^. The temperature-salinity curve of Ocean Reanalysis System 5 (ORAS5) matched our CTD data very well (see Supplementary Figs. S1 and S2). Therefore, we considered these data reliable and applied them to depict the buoyancy frequency profile. The value of *N* was estimated to be 1.0 × 10^−3^ Hz.

**Figure 5:**
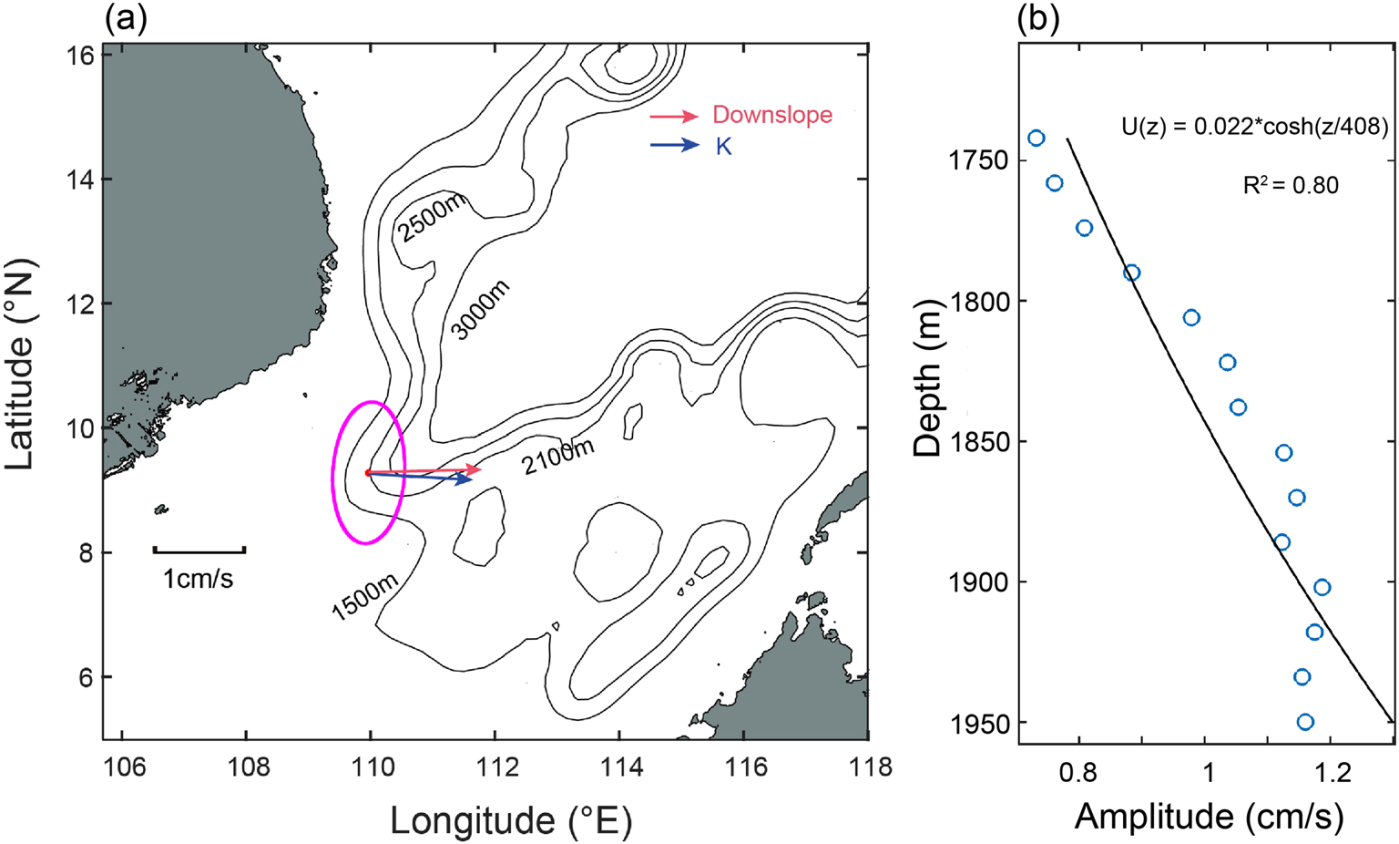
Characteristics of the TRWs in the southern SCS. (**a**) Standard deviation (STD) ellipses of 30–50 days bandpassed velocity of 1950 m at the mooring TJ-T. (**b**) First empirical orthogonal function (EOF) mode of the TRWs velocity anomaly profiles. The blue circles are observational velocity and the black solid line is the least squares fit of the first mode. The map was generated by MATLAB R2019b (https://ww2.mathworks.cn/).

Figure 5a shows the standard deviation ellipses of the 30–50 days band-passed fluctuations. The major axes of the ellipses demonstrate that the wave fluctuated almost parallel to the local isobath, which matches the characteristics of TRWs. The direction of K (minor axes of ellipses) appears offshore. Since the direction of the topographic gradient was uncertain and the standard deviation ellipses rotated counterclockwise with increased water depth, we estimated the angle *θ* to be ~ 10°. Using the given parameters, the frequency $\omega$ was calculated to be 2.76 × 10^7^ Hz, which corresponds well to ~ 40 days. Therefore, the 40−day fluctuations were proved to be TRWs.

The trapping length was conducted by the first-mode empirical orthogonal function (EOF) of the TRW velocity anomaly profiles with the application of the least squares fitting function^24^:

5 $$U\left( z \right) = {\text{A}}0 \times {\text{cos}}{\text{h}}\left( {z/h_{trap} } \right),$$

where *U*(z) denotes the first EOF mode of the TRW velocity anomaly and A0 is a constant. According to Hamilton^3^, the TRW wavelength is expressed as:

6 $$\lambda_{z} = \frac{NK}{f}.$$

To achieve a more precise fit, the time series was truncated from March 1 to July 1. The fitting result is displayed in Fig. 5b, revealing that ${\text{h}}_{\text{trap}}$ was approximately 408 m. It is worth noting that in this study, H > ${\text{h}}_{\text{trap}}$, which corresponds to a TRW wavelength of 109 km.

## Discussion

Previous studies have mostly attributed the energy source of TRWs to perturbations from upper layers^11,21,22,25^. However, we found little correlation between the burst of TRWs and surface variabilities, which suggests that this oscillation was not locally formed. To investigate the energy source, we used a ray-tracing model^10,25,26^.

**Figure 6:**
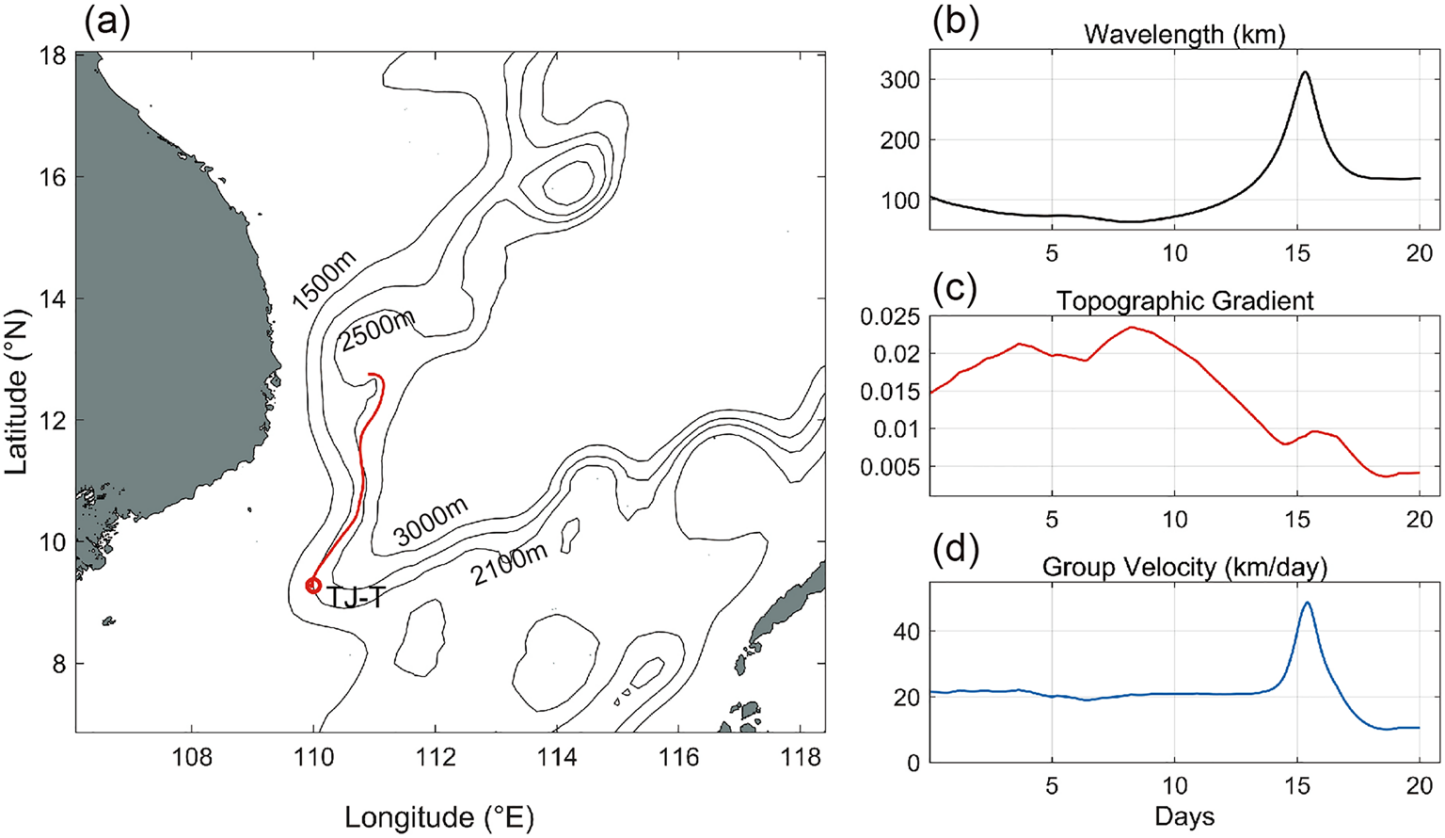
Inverted ray-tracing results of the TRWs in the southern SCS. (**a**) Wave ray (red line) from the ray-tracing model in the SCS. The bathymetry data are from GEBCO v2023 smoothed over 111 km (black lines). Theoretical wavelengths (**b**), topographic gradients (**c**), and group velocities (**d**) of the ~ 40 days TRWs along the ray. The maps were generated by MATLAB R2019b (https://ww2.mathworks.cn/).

The initial wavenumber pairs were given as (5.95 × 10^−5^ m^−1^, 6.25 × 10^−6^ m^−1^). The ray-tracing model was backward integrated for 20 days with a time step of 1 h. The calculated path (Fig. 6a) indicates that the wave originated from the northeast side of the mooring station, mainly following the isobaths, which is consistent with previous studies^21,23^. The group velocity followed the same trend as the wavelength but increased with the decreasing $\nabla {\text{H}}$ (Fig. 6b).

**Figure 7:**
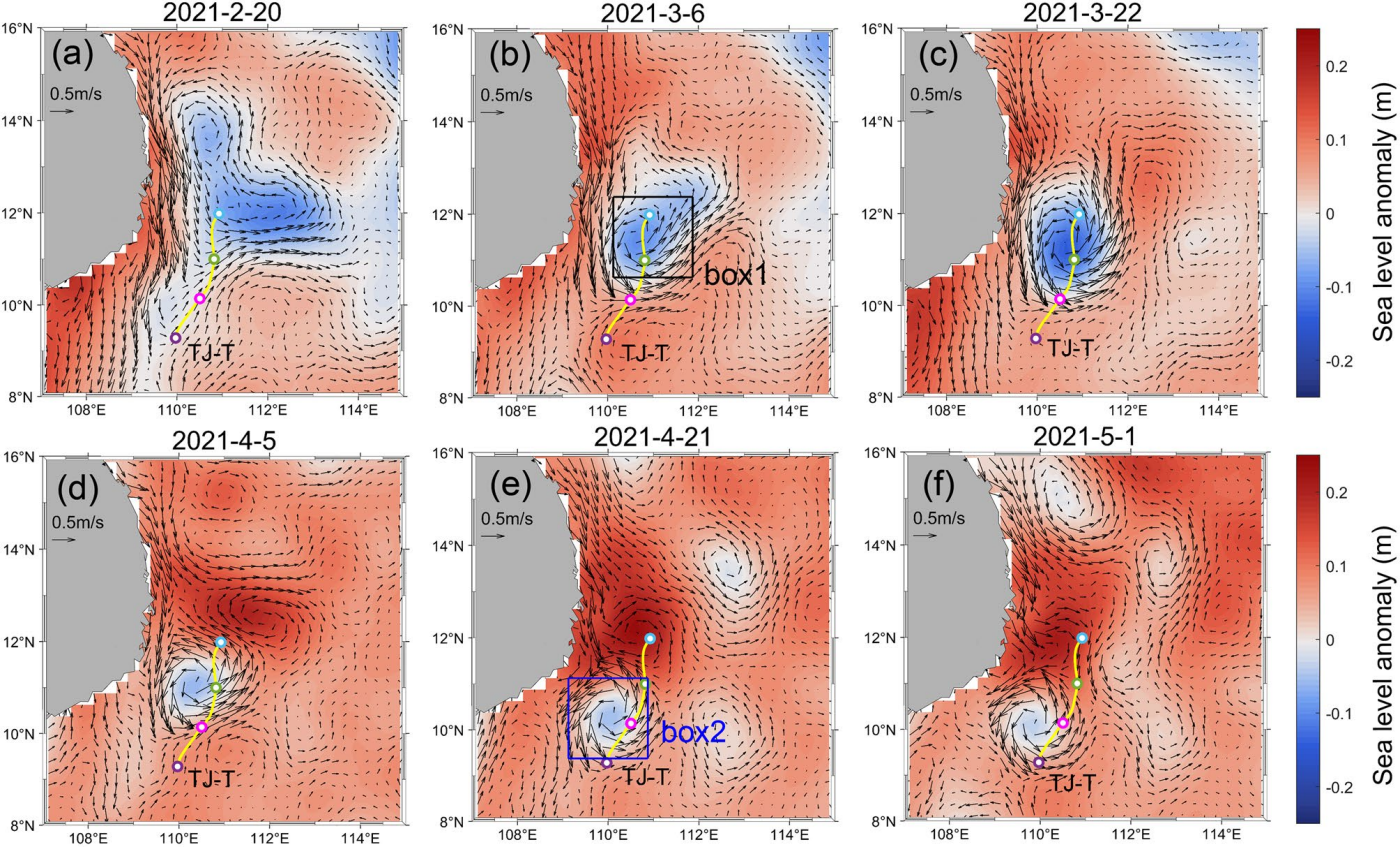
Sea level anomalies and geostrophic currents (vectors; units: cm/s) from (**a**) February 20, 2021 to (**f**) May 1, 2021 in the southwestern SCS. The yellow line indicates the ray. The purple point represents the location of the mooring TJ-T, and the pink, green and blue points represent the locations 5, 10 and 15 days before the TRWs appeared at the mooring TJ-T. Box 1 represents the region where the eddy initially formed near the ray path, while box 2 represents the region where the moving eddy was about to decay. The maps were plotted using MATLAB R2019b (https://ww2.mathworks.cn/) with M_Map (a mapping package, https://www.eoas.ubc.ca/~rich/map.html).

**Figure 8:**
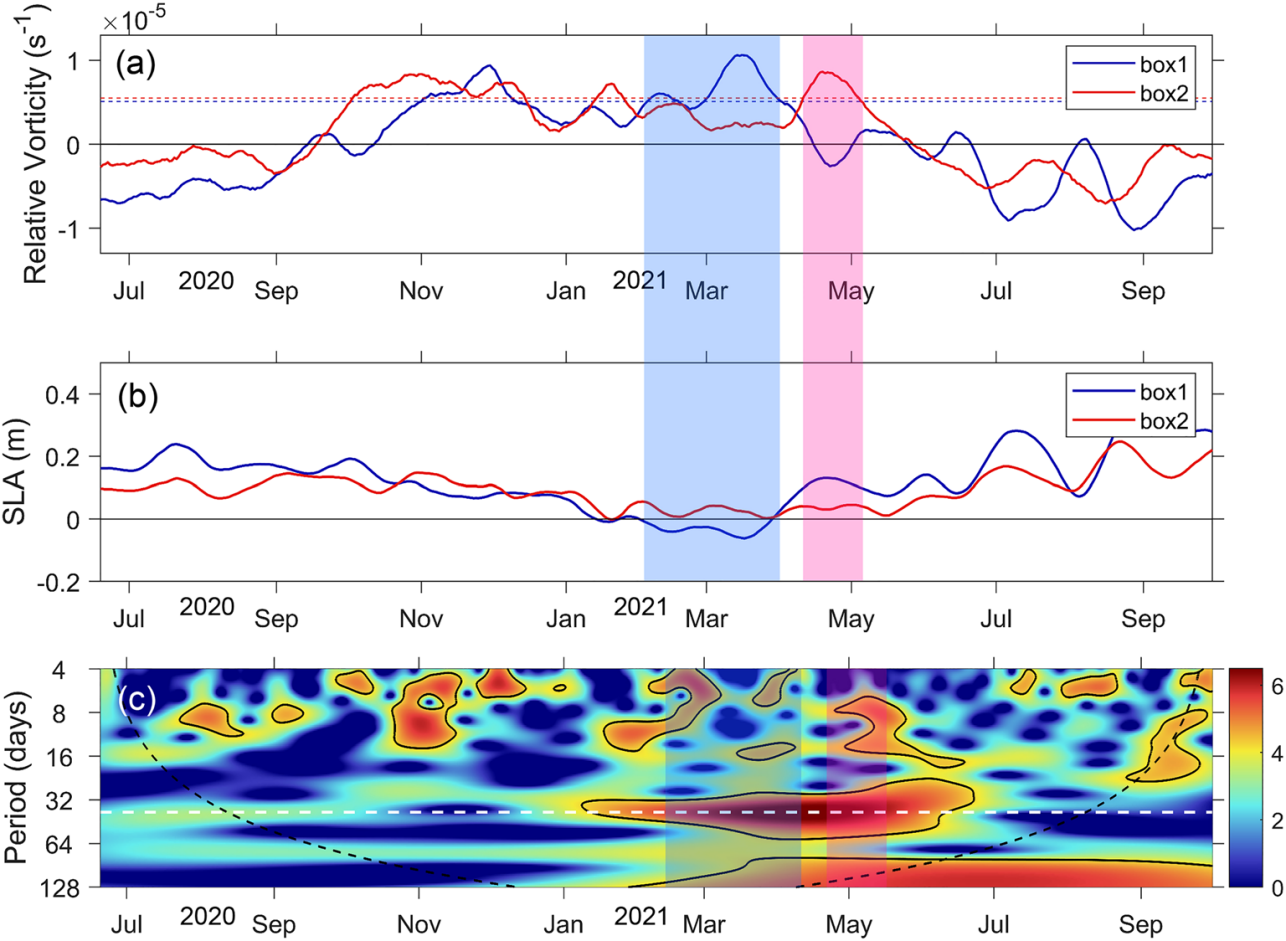
Time series comparison of relative vorticity and sea level anomaly in the eddy area and wavelet spectra of the mooring TJ-T in the southwestern SCS. (**a**) Time series of the average relative vorticity in box 1 (blue line) and box 2 (red line). The dashed lines present the mean value plus standard deviations, respectively; (**b**) mean sea level anomaly in box 1 and box 2; (**c**) wavelet spectrum of the observed meridional velocity of the mooring TJ-T. The blue and pink shadings in (**a**) and (**b**) represent the periods when the positive relative vorticity value is greater than the mean value plus the respective standard deviations. The shadings in (**c**) indicate the shadings in (**a**) and (**b**) but with additional respective time lags.

A cyclonic mesoscale eddy formed along the ray path off eastern Vietnam in March and lasted for almost 2 months (Fig. 7). It gradually weakened and propagated southwestward, with an anticyclonic eddy forming on its north side. We found that the burst of these mesoscale eddies took place approximately 10 days before notable TRWs were detected at the mooring station (Fig. 7). During this period, the TRWs could be precisely traced back to the eddy, suggesting that the eddy may serve as an energy source. Figure 8 shows the time series of the average surface relative vorticity ζ (ζ = ∂v/∂x − ∂u/∂y), sea level anomaly in box 1 and box 2, and wavelet spectrum of the meridional velocity at the mooring TJ-T. We hypothesized that TRWs are more likely to be triggered when the positive vorticity reaches relatively high value (shadings in Fig. 8), though this criterion may be somewhat stringent. The power density increased dramatically following the onset of eddies with a 10 − 15 days phase delay, corresponding to an approximately 0.23 m/s group velocity. Nevertheless, the cyclonic eddy in box 1 did not persist long enough to support TRWs until May. The fact that the eddy exactly traced the ray path of the TRWs observed at TJ-T suggests this moving eddy may continuously generate the TRWs. As shown in Fig. 7, the eddy was about to dissipate near the mooring TJ-T. We estimated the mean vorticity in box 2, which indicates that the active eddy in box 2 lasting until early May was able to continue supplying energy to TRWs. Previous studies have shown that the energy of the upper eddies propagates to the lower layer by adapting the potential vorticity to the changing height of the lower layers, generating TRWs^3,20,22^. In other words, energy is transferred downwards through pressure work^27,28^. Actually, it is important to note that the ray tracing model only approximately depicts the propagation path of the TRWs because it ignores the background circulation, non-linear process, and local energy generation in the real ocean. These factors can affect the wavelength and direction of the TRWs^23^, increasing the uncertainty of the estimations of the time difference. The area off Vietnam is identified to have high eddy kinetic energy in the SCS^29,30^, but it is unclear to what extent energy injection in this region may influence a broader range in terms of distance and depth. This research provides the first evidence that eddies in this area can affect deep currents. In addition, this is the first report of continuous eddy energy supply exciting TRWs when the eddy migration path coincides with the TRWs’ path. This likely represents a problem related to the forcing mechanism of TRWs caused by upper disturbances, distinguishing it from previous observational cases of TRWs.

However, several issues still need to be clarified. The surface eddies may not be the sole driving force in this case, as weak amplitudes of this band can be found at other times. This could be due to the contribution of deep flow instabilities^31^, but further investigation is not possible due to data limitations. In addition, the origin and generation of the TRWs need to be analyzed through a quantitative study of variations in the pressure work and baroclinic instability terms. Furthermore, the mechanism that how the moving eddy frequently affects the characteristics of TRWs requires further elucidation. To address these issues, more in-situ observations and models of high quality and higher vertical resolution in deep ocean are required in the future.

## Methods

### Observation data

The mooring TJ-T was situated on the continental slope of the southwestern South China Sea (9.28° N, 109.97° E) between June 20, 2020 and October 1, 2021, at a water depth of 2220 m. A 75 kHz Long Ranger Acoustic Doppler Current Profiler (ADCP) was installed at a depth of 1710 m, with a 30 min temporal resolution and 16 m vertical resolution, covering a 480 m water column (30 water bins). After eliminating invalid and substandard data, we obtained actual velocity coverage from 1742 to 2078 m. Additionally, missing values and data with ‘Percent Good’ values < 70 were replaced with spline interpolation. The actual observation depth of Conductivity Temperature Depth (CTD) was at 1730 m, with a 1-min temporal resolution.

### Downloaded data

The bathymetry data was sourced from the General Bathymetric Charts of the Oceans (GEBCO) version 2023, at a 15 arc-second spatial resolution (https://download.gebco.net/). To assess daily sea level anomaly and sea surface geostrophic velocities, data from a suite of altimetry satellites spanning from GEOSAT to Jason-3 was utilized (https://doi.org/10.48670/moi-00149). The altimeter data were processed and curated by CNES/CLS’s operational multi-mission production system. Additionally, we estimated the Brunt-Väisälä frequency using temperature and salinity profiles from the monthly mean of the Ocean Reanalysis System 5 (ORAS5), downloaded from https://doi.org/10.24381/cds.67e8eeb7.

### Ray-tracing model

Given the dispersion relations above, the path of TRW energy can be backtracked by applying a ray-tracing model^25^. Under the Wentzel-Kramers-Brillouin (WKB) approximation, the equations of the wave path are as follows^25^:

7 $$\frac{{d{\text{X}}}}{dt} = \frac{\partial \omega }{{\partial K}} = C_{g}$$

8 $$\frac{dK}{{dt}} = - \mathop \sum \limits_{i = 1}^{n} \frac{\partial \omega }{{\partial \gamma_{i} }}\nabla \gamma_{i}$$

where $\frac{{\text{d}}}{{\text{dt}}}=\left(\frac{\partial }{\partial {\text{t}}}\right)+{C}_{g}\cdot \nabla$ is the derivative following the wave group, X is the location of the wave group (i.e., the ray), K = (*k, l*) is the wavenumber vector, and ${C}_{g}$ is the TRW group velocity. ${\gamma }_{i}$ are the environmental parameters that cause refraction of the wave. In our research there are 3 parameters in total: *N* (the Brunt-Väisälä frequency), H (water depth), and $\nabla {\text{H}}$ (topographic gradient).

### Supplementary Information

Supplementary Information.

## Data Availability

The datasets used and analyzed during the current study are available from the corresponding author upon reasonable request.
