# Supplementary material for: Mesoscale eddy-strengthened deep-sea topographic Rossby waves in the southwestern South China Sea
Source: Sci Rep. 2024 May 18;14:11362. doi: 10.1038/s41598-024-62040-z (PMC11102490; doi:10.1038/s41598-024-62040-z)
Supplement: Supplementary file 1 — Supplementary Information. [file 41598_2024_62040_MOESM1_ESM.pdf]

Scientific Reports Supplementary material for:

Mesoscale eddy-strengthened deep-sea topographic Rossby waves in the southwestern South China Sea

Wenzhuo Wang[1], Zhifei Liu[1,*], Yulong Zhao[1], Baozhi Lin[1], Xiaodong Zhang[1], Jingwen Zhang[1], Jiaying Li[1], Junyuan Cao[1] & Hongzhe Song[1]

[1]State Key Laboratory of Marine Geology, Tongji University, Shanghai, China
*E-mail: lzhifei@tongji.edu.cn

This supplementary material contains two figures. Supplementary Figure S1 compares data from Ocean Reanalysis System 5 (ORAS5) and observed data from Conductivity-Temperature-Depth (CTD) at the mooring TJ-T. Supplementary Figure S2 shows the porfile of Brunt-Väisälä frequency *N* at the mooring TJ-T.

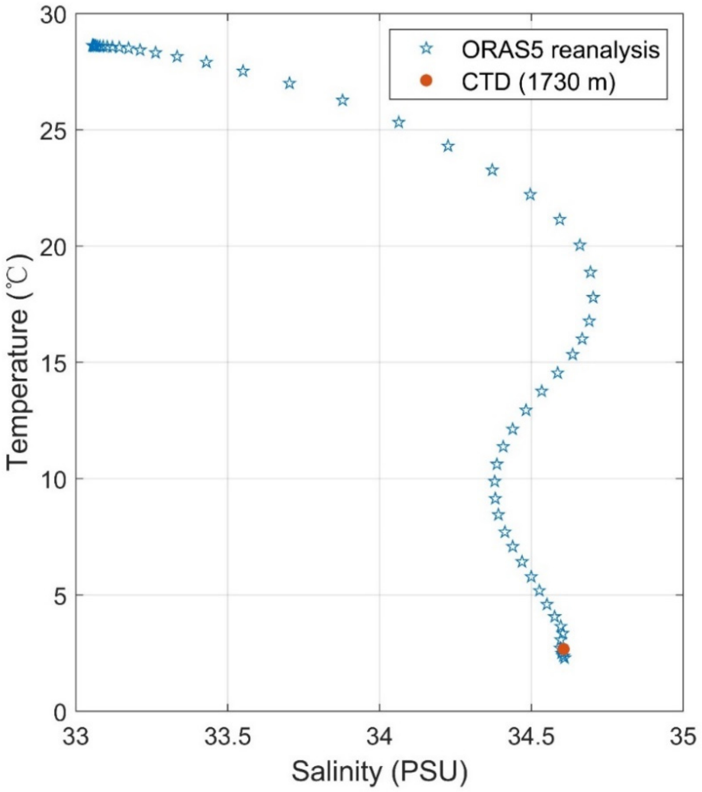

Supplementary Figure S1. Temperature-Salinity curve of ORAS5 (blue stars) from 0 m to1758 m depth and of CTD (orange point) observed at the mooring TJ-T at 1730 m in the southwestern South China Sea.

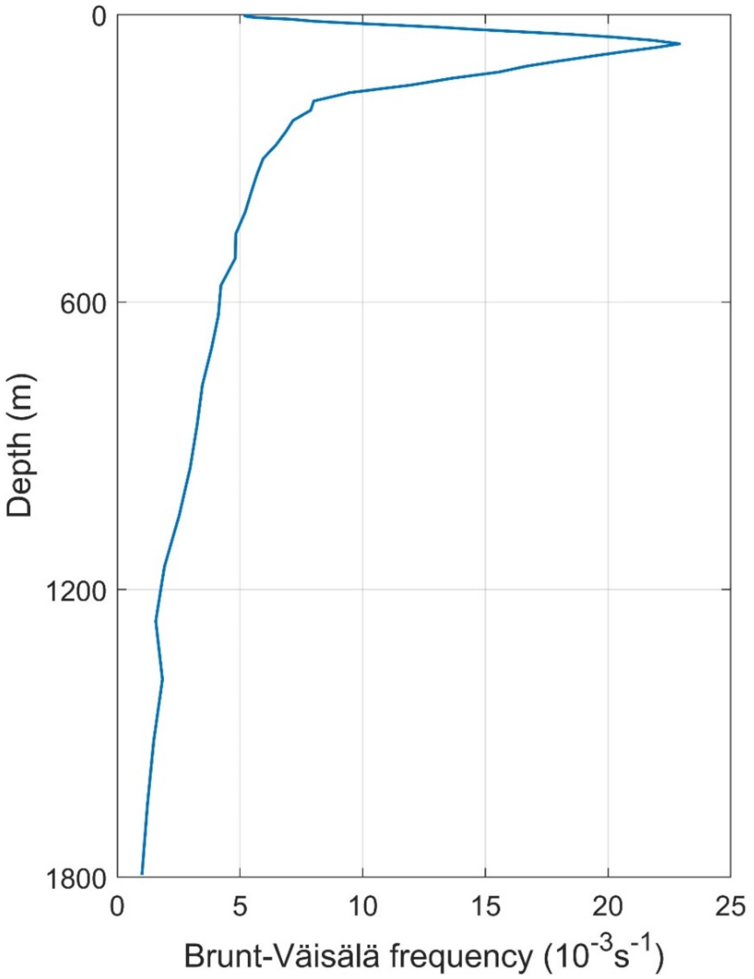

Supplementary Figure S2. Profiles of Brunt–Väisälä frequency N of ORAS5 at the mooring TJ-T in the southwestern South China Sea.
